# AvrRpm1 Missense Mutations Weakly Activate RPS2-Mediated Immune Response in *Arabidopsis thaliana*

**DOI:** 10.1371/journal.pone.0042633

**Published:** 2012-08-06

**Authors:** Karen A. Cherkis, Brenda R. S. Temple, Eui-Hwan Chung, John Sondek, Jeffery L. Dangl

**Affiliations:** 1 Department of Biology, University of North Carolina, Chapel Hill, North Carolina, United States of America; 2 Howard Hughes Medical Institute, University of North Carolina, Chapel Hill, North Carolina, United States of America; 3 Curriculum in Genetics and Molecular Biology, University of North Carolina, Chapel Hill, North Carolina, United States of America; 4 Department of Microbiology and Immunology, University of North Carolina, Chapel Hill, North Carolina, United States of America; 5 Carolina Center for Genome Sciences, University of North Carolina, Chapel Hill, North Carolina, United States of America; 6 Department of Pharmacology, University of North Carolina, Chapel Hill, North Carolina, United States of America; 7 Department of Biochemistry and Biophysics, University of North Carolina, Chapel Hill, North Carolina, United States of America; 8 Lineberger Comprehensive Cancer Center, University of North Carolina, Chapel Hill, North Carolina, United States of America; 9 R.L. Juliano Structural Bioinformatics Core Facility, University of North Carolina, Chapel Hill, North Carolina, United States of America; Universidad Nacional de La Plata, United States of America

## Abstract

Plants recognize microbes via specific pattern recognition receptors that are activated by microbe-associated molecular patterns (MAMPs), resulting in MAMP-triggered immunity (MTI). Successful pathogens bypass MTI in genetically diverse hosts via deployment of effectors (virulence factors) that inhibit MTI responses, leading to pathogen proliferation. Plant pathogenic bacteria like *Pseudomonas syringae* utilize a type III secretion system to deliver effectors into cells. These effectors can contribute to pathogen virulence or elicit disease resistance, depending upon the host plant genotype. In disease resistant genotypes, intracellular immune receptors, typically belonging to the nucleotide binding leucine-rich repeat family of proteins, perceive bacterial effector(s) and initiate downstream defense responses (effector triggered immunity) that include the hypersensitive response, and transcriptional re-programming leading to various cellular outputs that collectively halt pathogen growth. Nucleotide binding leucine-rich repeat sensors can be indirectly activated via perturbation of a host protein acting as an effector target. AvrRpm1 is a *P. syringae* type III effector. Upon secretion into the host cell, AvrRpm1 is acylated by host enzymes and directed to the plasma membrane, where it contributes to virulence. This is correlated with phosphorylation of Arabidopsis RIN4 *in vivo*. RIN4 is a negative regulator of MAMP-triggered immunity, and its modification in the presence of four diverse type III effectors, including AvrRpm1, likely enhances this RIN4 regulatory function. The RPM1 nucleotide binding leucine-rich repeat sensor perceives RIN4 perturbation in disease resistant plants, leading to a successful immune response. Here, demonstrate that AvrRpm1 has a fold homologous to the catalytic domain of poly(ADP-ribosyl) polymerase. Site-directed mutagenesis of each residue in the putative catalytic triad, His63-Tyr122-Asp185 of AvrRpm1, results in loss of both AvrRpm1-dependent virulence and AvrRpm1-mediated activation of RPM1, but, surprisingly, causes a gain of function: the ability to activate the RPS2 nucleotide binding leucine-rich repeat sensor.

## Introduction

*Pseudomonas syringae* is a Gram-negative phytopathogen that utilizes various biochemical means, including analogous enzymatic activity or molecular mimicry of host proteins, to block or bypass the plant immune system. To achieve this, each *P. syringae* strain injects a suite of effector proteins into host cells using a type III secretion system. The type III secretion system is shared by many Gram-negative pathogens of plants and animals that use effector proteins to subvert host cell physiology and bypass defenses [1]–[3]. Plants have evolved an elaborate intracellular detection system to recognize effectors that attempt to block or dampen MAMP-triggered immunity (MTI), and reinitiate the blocked immune response [4]. Several well-studied nucleotide binding leucine-rich repeat (NB-LRR)-dependent responses to effectors are mediated by indirect recognition of effector action on a host target, as described by the Guard Hypothesis [4], [5]. In this model effector targets functions as a molecular lure or ‘guardee’, and a specific NB-LRR protein functions as a ‘guard’ [6]–[9]. Upon biochemical manipulation of the guardee by an effector protein, the NB-LRR protein is activated [4], [5], [10], leading to a successful immune response. In the absence of the corresponding NB-LRR, manipulation of the guardee can contribute to the virulence activity of the effector [4], [7].

This work focuses on the characterization of *Pseudomonas syringae* type III effector protein AvrRpm1. AvrRpm1 function requires consensus fatty acid acylation sites including the myristoylation site of Gly2, likely followed by a subsequent palmitoylation site at Cys3 [11]. Once localized at the plasma membrane, AvrRpm1 associates with RIN4, and, by an unknown mechanism, triggers its phosphorylation [7]. RIN4 phosphorylation is presumed to activate RPM1 and consequent downstream disease resistance responses. This model has been experimentally validated for a second, sequence diverse type III effector, AvrB, which targets the same RIN4 sub-domain targeted by AvrRpm1 to activate RPM1 [12]. In the absence of RPM1, AvrRpm1 [13] and AvrB [14] can contribute to overall pathogen virulence. Moreover, in the absence of both RPM1 and RIN4, AvrRpm1 still contributes to virulence [15], strongly suggesting that additional targets for AvrRpm1 exist in Arabidopsis. Targeting of RIN4 by two additional *P. syringae* effectors, AvrRpt2 [16]–[18] and HopF2 [9] suggest that RIN4 is a point of convergence in the arms race between pathogen effectors and critical host defense machinery [19].

Even though type III effectors are the main contributors to the overall virulence of a phytopathogen, their diverse biochemical functions in the host cell have only recently started to be dissected; these include E3 protein ligase, phosphothreonine lyase, and ADP-ribosyl transferase activities [20]–[23]. Determination of molecular functions for type III effectors is complicated by their relatively low conservation at the primary amino acid sequence level to proteins of known biochemical function, suggesting convergent evolution onto structures that modulate eukaryotic signaling pathways [24], [25]. Therefore, we used tertiary structure prediction in order to gain insight into AvrRpm1 function. We found that AvrRpm1 consists of the fold from the catalytic domain of poly(ADP-ribosyl)polymerase-1 (PARP-1).

PARPs belong to a large family of proteins that contain additional domains beyond the canonical catalytic domain [26]. PARPs undergo self-modification by addition of ADP-ribose moiety(s) from NAD or function analogously on other targets. The addition of poly(ADP-ribose) (PAR) is reversible by poly(ADP-ribose) glycohydrolases (PARGs) [27]. Poly(ADP-ribose) (PAR) can be toxic, often leading to inflammation, ischemia, and eventually cell death in mammalian systems [28]. Nudix O-acetyl-ADP-ribose hydrolases are responsible for the breakdown of free PAR within the cell [29]. The Arabidopsis genome encodes both PARGs and Nudix hydrolases, and both have been implicated in immune responses [30], [31]. More generally, ADP-ribosylation of target proteins by toxins and type III effectors results in the manipulation of host signaling and defense machinery in both plant and animals, as evidenced by the structurally related proteins Diphtheria toxin from *Corynebacterium diphtheriae*, ExoS from *Pseudomonas aeruginosa*, and HopF2 from *P. syringae*, and the structurally unrelated HopU1 [22], [32]–[35].

We demonstrate that the AvrRpm1 family of type III effectors shares the PARP catalytic fold, including key catalytic and structural components of PARP such as the catalytic triad H862-Y907-E998, which typically facilitates the ribosylation reaction. We use mutagenesis and functional tests to demonstrate that the conserved putative catalytic residues are required for AvrRpm1 to either elicit an RPM1-dependent immune response or contribute to virulence on a susceptible host. Furthermore, and quite intriguingly, we show that putative catalytically inactive AvrRpm1 inhibits the growth of *P. syringae* pathovar (pv.) *maculicola* on disease susceptible plants. This growth inhibition is dependent on activation of the NB-LRR protein RPS2. These findings support previous work suggesting that over-expressed AvrRpm1 has an ‘off target’ ability to trigger an RPS2-mediated defense response, and that RIN4 is not the only target for AvrRpm1 [15], [36], [37].

Despite our inability to demonstrate enzymatic activity, due to inherent instability of purified AvrRpm1, our results collectively support the hypothesis that AvrRpm1 is a PARP-type ADP-ribosylating protein. Our data provide a starting point for identification of a substrate for AvrRpm1 and for the definition of how that substrate contributes to RIN4 phosphorylation and inhibition of host defense. Our results also highlight the need for further understanding of the complex relationship between RPM1, RPS2, RIN4 and RIN-like proteins that may also be functionally relevant in this system.

## Materials and Methods

### Creation of the Homology Models

The models were generated by querying the BioInfoBank Institute's metaserver where we initially were able to detect homology with the catalytic domain of PARP-1. We compared sequence alignments generated with ClustalX, using the programs InSIGHTII, Accelrys Software Inc., and MODELLER [38]–[41]. We used PDB IDs: IUK0, IGS0, 1A26, and 3GJW as templates to generate a structural map for which we could align the AvrRpm1 sequences. The model for the *Psm* allele was then evaluated for fitness using the Verify 3D application in InSIGHTII.

### Generation of AvrRpm1 mutants and *P. syringae* strains

Missense mutations for AvrRpm1 were generated by gene splicing [42]. The external PCR primers are Gateway™ compatible so that a common entry vector product could be used for the generation of multiple destination vectors. *Pto* DC3000, *Psm* CR299 carried the engineered missense mutations *in trans* on the pDLTrp plasmid, a Gateway™ compatible derivative of the pBBR1MCS vector [43] that uses a constitutively active tryptophan promoter. Missense alleles of AvrRpm1 were expressed in *Pst* DC3000 as fusions to *Δ79avrRpt2* as previously described [44]. An avirulent *P. fluorescens* (*Pf0*) strain that has been engineered to carry a stable integration of the *hrp/hrc* cluster as previously described [45] was transformed with different combinations of the plasmids pVSP61 carrying *avrRpt2* [46], [47] or the pDLTrp plasmid mentioned above carrying either wild type *avrRpm1* or the missense mutations.

### Electrolyte leakage, bacterial growth and translocation assay conditions

Electrolyte leakage assay has been described [6] and modified to include 4 leaf discs in 6 mL of water. Bacterial growth in leaves was measured by inoculating 10^6^ cfu/mL into the leaves of 4–5 week old plants. Leaf discs were extracted and ground in 10 mM MgCl_2_ and serially diluted to measure bacterial numbers on the day of infiltration as well as 3 days post infiltration (3 dpi). ANOVA and a Tukey's post-hoc analysis were performed on the 3pi data using the JMP ® Genomics software suite, SAS Institute Incorporated © 2012 to determine if there was a statistically significant difference among the growth levels of the various strains. Bacterial growth in seedlings was measured by dip inoculation as previously described [48]. Briefly, an inoculum of 10^5^ cfu/mL was made for *Pto* DC3000 carrying either an empty vector or *avrRpm1* with missense mutations. Bacterial growth was measured on the day of inoculation as well as 3pi. Translocation assays were performed by inoculation of 4–5 week old plants with 5×10^7^ cfu/mL on one side of the leaf. Leaves were collected and photographed 20 hpi.

### Protein accumulation and immunoblot assay

For accumulation of proteins in plant tissue, leaf samples were ground in extraction buffer containing 20 mM Tris pH 8.0, 150 mM NaCl,1% Triton X-100, 1 mM EDTA pH 8.0, 0.1% SDS, 10 mM DTT Plant Protease Inhibitor Cocktail from Sigma-Aldrich. Ground tissue was centrifuged for 20 minutes at 20,000× g. Supernatant was quantified by Bradford analysis, subjected to SDS-PAGE and immunoblot analysis.

### AvrRpm1/AvrRpt2 RIN4 competition assay

*Pfo* strains described in the methods section for generation of AvrRpm1 mutants were infiltrated at 10^8^ cfu/mL into 4-week-old plants. Two leaves were collected for each time point and tissue was harvested as described above. Extracts were subjected to immunoblot analysis and probed with an α-RIN4 antibody generated from a highly specific and antigenic peptide of RIN4.

### Ribosylation Assay

Seedlings of either *rpm1* or *Dex::AvrRpm1-HA* in *rpm1* genotypes were sparsely sown and grown on MS plates for 14 days [49]. The seedlings were then sprayed with a solution of 25 µM dexamethasone (Sigma) and 25 nM biotinylated NAD (Trevigen). The protein was extracted using the protocol described in the protein accumulation and immunoblot assay methods. Duplicate preparations were made and one set was treated with phosphodiesterase type I (Sigma) in 110 mM Tris pH 9.0, 110 mM NaCl and 15 mM MgCl_2_ [50]. The extracted protein was subjected to immunoblot analysis and probed using pre-conjugated α-streptavidin (Thermo). For agrobacterium-mediated transient ribosylation assay we followed the protocol established in [12]. We then followed the protocol outlined above for labeling with biotinylated NAD and phosphodiesterase type I treatment.

## Results

### Identification of conserved structural homology and a putative PARP catalytic triad in AvrRpm1

**Figure 1:**
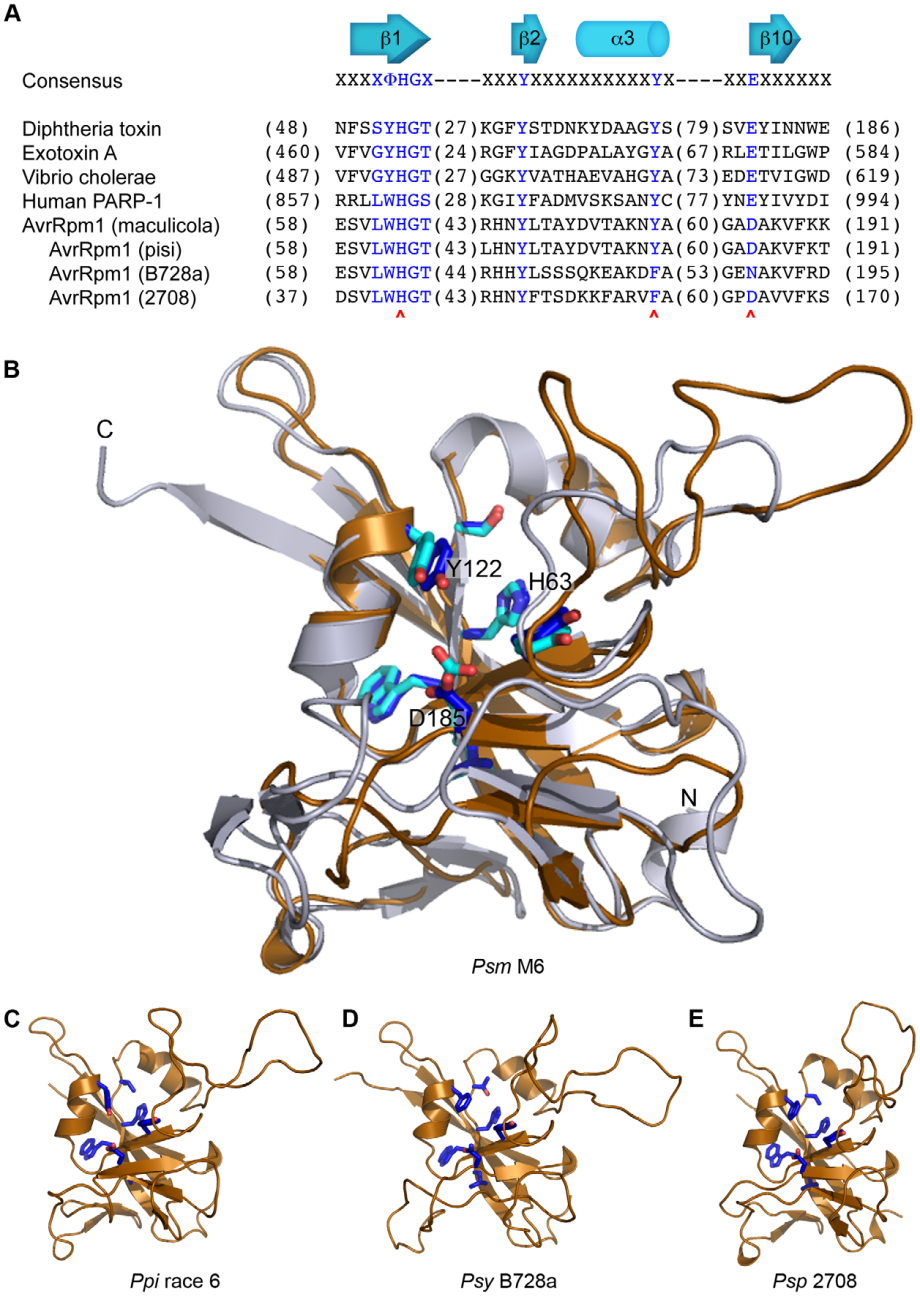
AvrRpm1 exhibits structural homology to the catalytic domain of Poly-ADP-ribosyl polymerase (PARP). (**A**) Sequence alignment of DT family ADP-ribosylating proteins [35] and the four AvrRpm1 family proteins illustrating key regions of conservation. Secondary structure for each region is shown above. Highly conserved residues are highlighted in blue. Red carets denote the catalytic triad of PARP. (**B**) Homology model of the AvrRpm1 reference allele (copper) from *P. syringae* pv. *maculicola* M6 (*Psm* M6) with the catalytic domain of Poly-ADP-ribosyl polymerase 1 (PARP-1; PDB ID: 3GJW) (silver). The side chains for residues highlighted in (**A**) are denoted by dark blue (AvrRpm1) and light blue (PARP-1). Residues in the catalytic triad are labeled according to AvrRpm1. “N” and “C” represent the amino- and carboxy-terminus of the protein respectively. Independent homology models for the remaining three AvrRpm1 family members from (**B**) *P. syringae* pvs. *syringae* B728a (*Psy* B728a), (**C**), *pisi* race 6 (*Ppi* race 6) (**D**), and *phaseolicola* 2708 (*Psp* 2708).

We generated a computational homology model for AvrRpm1 to identify conserved structural domains shared with proteins of known function. After removing the first 30 residues, which are predicted to be disordered, we input the remaining AvrRpm1 amino acid sequence into the BioInfoBank Institute's metaserver [51]. The highest-ranking outputs for predicted homologous folds from the aggregated databases were to various catalytic domains of poly(ADP-ribosyl)polymerase (PARP) [26], [52]. PARP is a member of the larger family of Diphtheria toxin-like ADP-ribosyl transferases [35], [53], [54]. The catalytic domain of these proteins can be broken down into three regions (Figure 1A). The N-terminal region 1 is a span of primarily conserved residues highlighted by an aromatic residue (Figure 1A, denoted with Φ) followed by the first catalytic triad member H63 (in AvrRpm1; all residues noted refer to the allele from *Psm* M6, GEN BANK ID AF359557.1 unless stated otherwise) and a glycine (G64). We also noted the presence of a conserved leucine (L62) preceding this region and a serine or threonine (T64) at its end in the majority of the Diphtheria toxin-like ADP-ribosyl transferase proteins. The centrally located region 2 is denoted by a pair of tyrosine residues Y111 and Y122 separated by ten amino acids, where Y122 corresponds to the second member of the catalytic triad. The C-terminal region 3 contains the third catalytic triad residue, glutamate, or in the case of AvrRpm1 (*Psm* M6) aspartate (D185). Mutation of the glutamate residue to an aspartate did not abolish PARP-1 activity, but rather altered the *in vitro* kinetics [55]. The overall sequence identity between the catalytic domain of PARP-1 and AvrRpm1 is relatively low, however these regions and the relative spacing between them are conserved.

PARP-1 is a multi-domain protein [52], yet our homology model demonstrates that conservation with AvrRpm1 is limited to the catalytic domain. Hence, the model generated includes 70% of AvrRpm1, but only 16% of PARP-1. Our model begins at residue 49 of AvrRpm1 and extends until residue 203. The validity of our model was assessed using Verify3D, a program that compares the model generated and its own amino acid sequence [56]. The normalized average Verify3D score for the all residues in the model was 0.26, with a typical score around 1.0 for crystal structures and a typical score around 0.0 for incorrect folds. On average, scores above 0.10 reflect models with some structural validation. While there are loop regions that could not be accurately modeled, it is important to note that the core fold is predicted to be conserved between the two proteins (Figure 1B). These loop regions and regions at the amino- and carboxy-terminus of the model represent local minima in Verify3D score while regions spanning the core fold represent local maxima in the Verify 3D score and for these reasons we are confident in our model build. Models were also generated for each of the remaining AvrRpm1 family members: from *P. syringae* pvs. *pisi* race 6 (*Ppi* race 6), *syringae B728a* (*Psy* B728a), and *phaseolicola 2708* (*Psp* 2708) (AJ251482.1, [57], AAY35802.1 [58], and *Ps* pv. *phaseolicola* 2708 (unpublished) respectively) using the same PARP-1 templates (PDB IDs: IUK0, IGS0, 1A26, 3GJW) [59]–[62]. The spacing identified using the original metaserver output for the *P. syringae* pv. *maculicola* AvrRpm1 allele, as well as an amino acid sequence alignment for the four additional AvrRpm1 alleles (Figure S1), was used to create the remaining models (Figure 1C–E).

The *Psm* M6 and *Ppi* race 6 AvrRpm1 alleles share the highest identity, while the *Psy* B278a and *Pph* 2708 alleles are more divergent. Each AvrRpm1 family member, except that from *Psy* B728a, returned a structural match to either the PARP-1 catalytic domain (*Ppi*) or to PARP-12 and -15, smaller isoforms belonging to the PARP superfamily that contain only the catalytic domain (*Pph* 2708). We believe that the various programs aggregated in the metaserver were unable to identify a similar match for the *Psy* B728a allele due to a seven-residue deletion that occurs between regions 2 and 3. This deletion alters the position of the third putative catalytic triad residue (Figure 1A and S1).

### Putative PARP catalytic triad residues are required for activation of RPM1 by AvrRpm1

**Figure 2:**
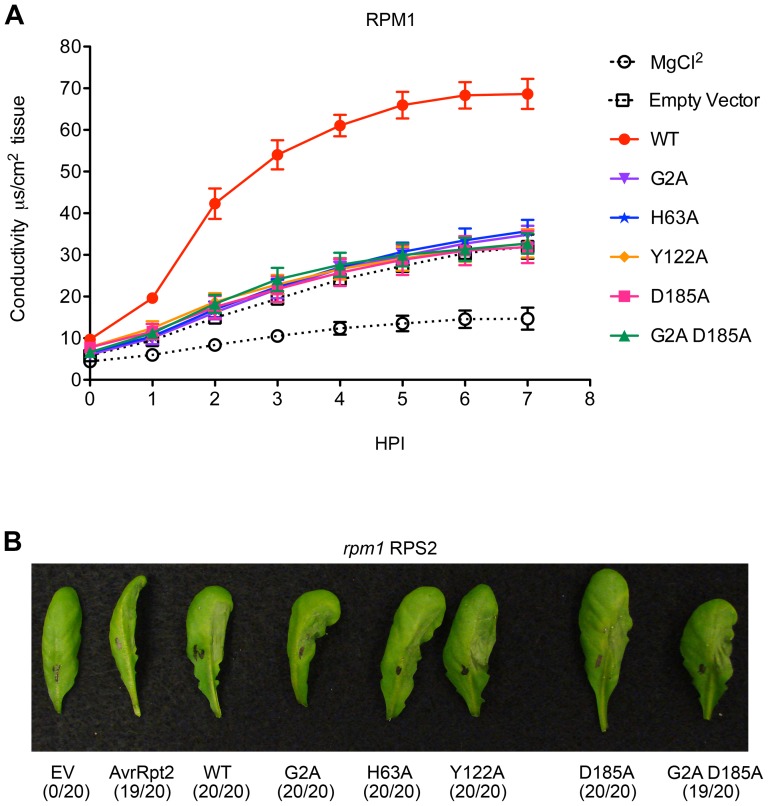
Missense mutants of AvrRpm1 do not elicit an RPM1-mediated hypersensitive response, but can be translocated. (**A**) Four week old Col-0 plants were hand inoculated with 5×10^7^ cfu/mL *Pto* DC3000 carrying either an empty vector or *avrRpm1* with missense mutations eliminating localization to the membrane (G2A) [11], to the putative catalytic triad (H63A, Y122A, and D185A) and a double mutant (G2A D185A) and assayed for the ability to promote electrolyte leakage via RPM1-mediated hypersensitive response (HR) (see Methods). Error bars represent 2× SEM. (**B**) Five week old *rpm1 RPS2* plants were infiltrated with 5×10^7^ cfu/mL *Pto* DC3000 carrying missense mutations of *avrRpm1* cloned to produce fusion proteins with Δ79*avrRpt2*. The ability to elicit an RPS2-mediated hypersensitive response was assayed at 20 hours post inoculation (HPI). Leaf counts (HR positive/total inoculated) are displayed under representative leaves.

Identification of a putative catalytic triad (H63-Y122-D185; Figure 1A) via homology modeling guided our introduction of missense mutations and subsequent functional tests following conjugation of mutant genes into *Pto* DC3000 (Methods). We assayed each of the three missense mutations (H63A, Y122A, D185A) for their ability to elicit AvrRpm1-dependent activation of RPM1 as measured by cellular electrolyte leakage, a proxy for HR cell death (Figure 2A). We found that each of the missense mutations was compromised in their ability to trigger RPM1-mediated HR, comparable to a previously characterized loss of function, mislocalization mutant G2A [11]. We also assayed for the ability of the missense mutations to trigger RPM1-dependent growth restriction of *Pto* DC3000 in wild-type plants (Col-0) [63]. We found that *Pto* DC3000 carrying the missense mutations were, surprisingly, unable to grow (Figure S2). One interpretation of this result is that these missense alleles retain the ability to initiate RPM1-dependent growth restriction, but not HR. However, data subsequently presented complicate this overly simple conclusion, and offer a clearer interpretation. To ensure that the AvrRpm1 missense alleles were not merely compromised in their ability to traverse the type III secretion system, we cloned each loss of function mutant as a fusion protein to truncated AvrRpt2 effector protein lacking the N-terminal 79 amino acids required for its own translocation [44]. These constructs were conjugated into *Pto* DC3000 and infiltrated into leaves of plants lacking RPM1, but expressing functional RPS2. These fusion effector proteins thus rely on the native type III secretion signals from AvrRpm1 for delivery into the host cell, but on the activity of Δ79AvrRpt2 to initiate RPS2-dependent HR. Each of the missense mutations was translocated via the type III secretion system (Figure 2B), an indication that the proteins are both expressed and stably accumulate to levels necessary for delivery into the host.

### Putative PARP catalytic triad residues are required for the virulence function of AvrRpm1

**Figure 3:**
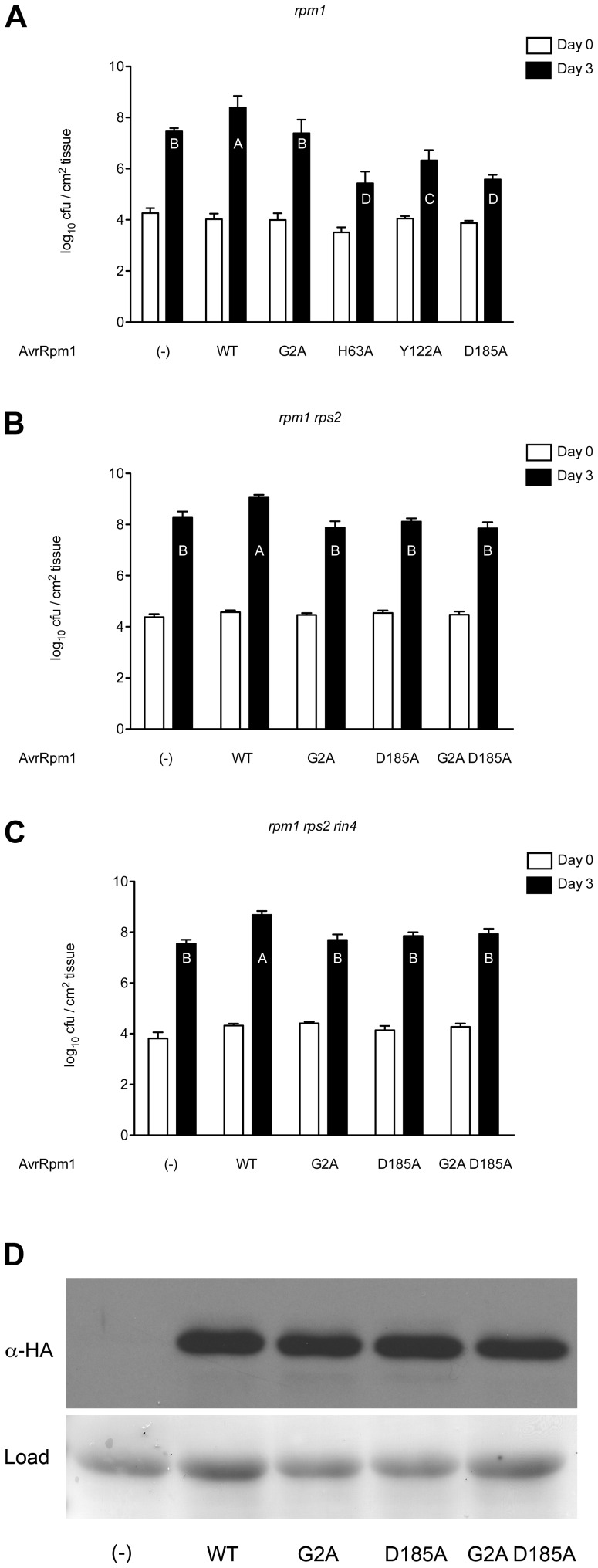
Putative catalytic triad residues are required for AvrRpm1 virulence that is inhibited via weak activation of RPS2-mediated disease resistance. (**A–C**) Growth of *Psm* CR299, a derivative of *Psm* M2 that carries an insertion in *avrRpm1* [13] was complemented in *trans* with plasmids expressing wild type AvrRpm1 and missense mutations as noted. Four week old *rpm1* (**A**), *rpm1 rps2* (**B**) or *rpm1 rps2 rin4* (**C**) plants were inoculated with 10^6^ cfu/mL and samples were collected on day 0 and day 3. Error bars represent 2× SEM. An analysis of variance (ANOVA) was performed among the day 3 samples followed by Tukey's post-hoc analysis (α = 0.05) with significance groups indicated by letters on the graph. (**D**) Immunoblot assay for accumulation of the wild type and mutant AvrRpm1 proteins at 3 days post inoculation for strains used in (**B**) and (**C**).

Each AvrRpm1 missense mutation was tested for its virulence [13]. AvrRpm1 missense mutations were expressed in *P. syringae pv maculicola* (*Psm*) strain M2 CR299, which carries an insertion in *avrRpm1* that disables this gene (CR299; [13]) (Figure 3A). *Psm* M2 CR299 carrying a wild type copy of *avrRpm1 in trans* grew at least ten-fold more than either *Psm* M2 CR299, or an isogenic strain that can deliver the mislocalized AvrRpm1 G2A missense mutant [11]. Each of the putative AvrRpm1 catalytic triad missense mutations was also compromised for virulence mediated by AvrRpm1. In fact, the expression of these putative catalytic triad mutants inhibited the growth of *Psm* M2 CR299 to a higher extent with respect to CR299 or to CR299 complemented with the localization AvrRpm1_G2A_ mutant (Figure 3A). To determine if plasma membrane localization was required for this surprising phenotype, we tested the virulence activity of an AvrRpm1 double mutant in both the putative catalytic activity and localization/myristoylation (AvrRpm1_G2A D185A_). We found that this strain grew to levels equal to *Psm* M2 CR299 expressing the mislocalized missense mutation G2A (Figure S3). These data suggest that the missense mutations must be properly localized inside the host cell in order to inhibit the growth of *Psm* M2 CR299.

### Missense mutations of AvrRpm1 proteins are perceived by the NB-LRR protein RPS2

Given the surprising result that *Psm* M2 CR299 strains expressing missense alleles in the putative catalytic triad of AvrRpm1 grew significantly less on susceptible *rpm1* hosts than controls, we wanted to investigate the mechanism responsible for this effect. We generated two plausible hypotheses (1) AvrRpm1_D185A_ binds its nominal target, or a new target, in an altered manner, causing sufficient target perturbation to activate an NB-LRR protein other than RPM1 to fire at low levels that are sufficient to limit pathogen growth; (2) AvrRpm1_D185A_ is able to bind its nominal target, or a new target, in a manner that sequesters this target from other type III effector proteins delivered by *Psm* M2 CR299, preventing them from effectively contributing to that strain's virulence. We assayed the ability of *Psm* M2 CR299-derived strains carrying the AvrRpm1 missense alleles to grow on plants that are null for both the RPM1 and RPS2 NB-LRR disease resistance proteins (*rpm1 rps2*), because RPS2 is an explicit candidate for weak recognition of AvrRpm1 [36]. In fact, *Psm* M2 CR299 expressing AvrRpm1_D185A_ grew as well as the loss of function mislocalization allele, AvrRpm1_G2A_ in leaves of *rpm1 rps2*. Thus, the ability of AvrRpm1_D185A_ expression to inhibit the growth of *Psm* M2 CR299 is due to weak activation of RPS2 that is insufficient to trigger macroscopic HR (Figure 2A and 3B). We observed the same growth patterns of *Psm* M2 CR299 expressing the AvrRpm1 missense alleles on susceptible *rpm1 rps2 rin4* plants (Figure 3C). Each of the AvrRpm1 missense mutations used in this assay accumulated normally in *P. syringae* (Figure 3D) and, as noted above, was translocated (Figure 2B). Together, these results demonstrate that the ability of the AvrRpm1_D185A_ to restrict growth of an otherwise virulent pathogen is dependent on its myristoylation and localization at the plasma membrane, and its ‘off-target’ perception there by RPS2.

### Missense alleles of AvrRpm1 do not show increased interference with AvrRpt2 cysteine protease activity on RIN4

**Figure 4:**
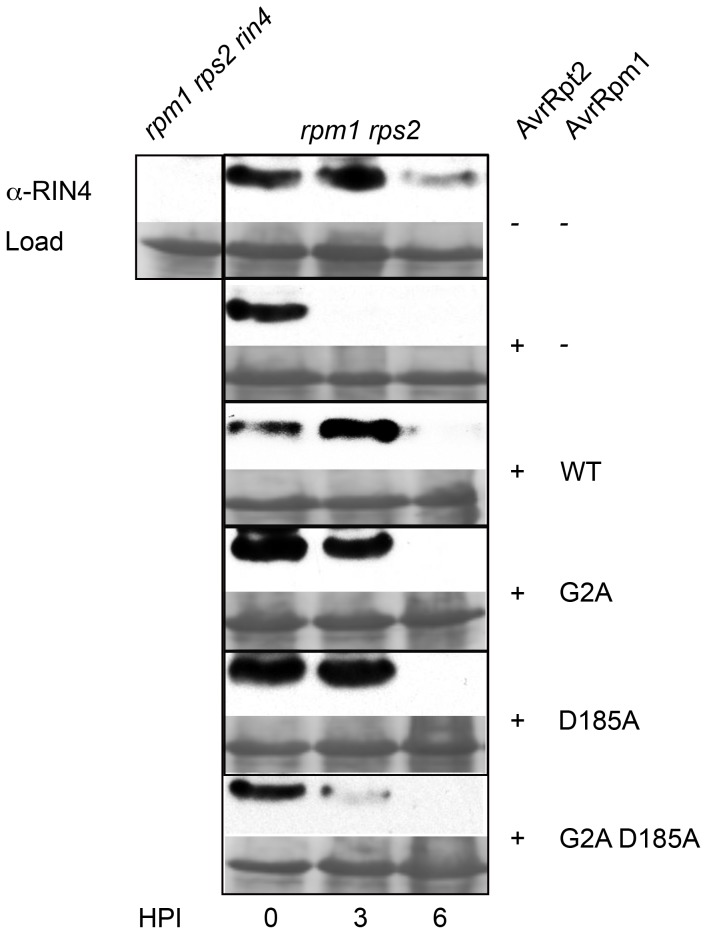
AvrRpm1 mutants do not exhibit increased interference with AvrRpt2-mediated cleavage of RIN4. *Pfo* expressing wild type AvrRpt2 and either wild type or AvrRpm1 missense mutations *in trans* was infiltrated into leaves of 4-week-old *rpm1 rps2* plants at 10^8^ cfu/mL. Samples were collected over a time course (as indicated) and probed for the presence of RIN4 as an output of AvrRpt2 function.

The type III effector AvrRpt2 functions as a cysteine protease that directly interacts with RIN4, cleaving it at N- and C-terminal RCS (*R*IN4 *c*leavage *s*ites) resulting in rapid degradation of the remaining RIN4 fragments [17], [18], [64]. One hypothesis to explain the results reported above is that AvrRpm1 prevents binding and cleavage of RIN4, or a RIN4-like substrate, by AvrRpt2. In this model, this blockade of the proposed substrate's ability to interact appropriately with RPS2 would lead to ectopic RPS2 activation in the same manner that RIN4 is genetically required to negatively regulate an otherwise lethal activation of RPS2 [6], [15]. Thus, AvrRpm1 and its missense alleles were tested for their ability to directly inhibit the ability of AvrRpt2 to cleave RIN4. Each AvrRpm1 missense mutation was expressed *in trans* together with AvrRpt2 in *Pseudomonas fluorescens*, a non-pathogen engineered to carry a competent type III secretion apparatus [45]. The ability of AvrRpt2 to cleave RIN4 leading to the overall disappearance of RIN4 over time was assayed via western blot analysis using native RIN4 antisera (Figure S4). Neither wild type AvrRpm1 nor the missense alleles consistently inhibited the cleavage and clearance of RIN4 by AvrRpt2 by 6 hours post-infection (Figure 4), consistent with the lack of effect of RIN4 on the growth suppression phenotype displayed by the AvrRpm1 missense alleles. However, we reproducibly detected attenuation of cleavage and clearance of RIN4 by AvrRpt2 in the presence of wild type AvrRpm1, mislocalized AvrRpm1_G2A_ or the putative non-functional AvrRpm1_D185A_. The double mutant AvrRpm1_G2A D185A_ reproducibly lacked this ability. We speculate that this attenuation is due to weak inhibitory activity of mislocalized, but functional, AvrRpm1_G2A_ on RIN4 [11], and a similar inhibitory activity of properly localized, but non-functional, AvrRpm1_D185A_ on RIN4. The lack of attenuation of RIN4 cleavage by AvrRpt2 observed for AvrRpm1_G2A D185A_ can be explained by the combined loss of function of the two single mutants. In sum these data do not eliminate the possibility that the AvrRpm1 missense alleles exhibit their novel phenotype via binding to another RIN4-related target more tightly than does the wild type AvrRpm1, leading to a previously undefined activation of RPS2 and subsequent pathogen growth suppression.

### AvrRpm1 may function as a molecular mimic of ADP-ribosyl transferases

We have tried to ascertain whether AvrRpm1 functions as an ADP-ribosyl transferase using established assays [22]. However, we were unable to successfully purify AvrRpm1 to homogeneity as a soluble and folded protein. We tested the ability of AvrRpm1 to catalyze a ribosyl-transferase reaction using biotinylated nicotinamide adenine dinucleotide (NAD) as a substrate in a dexamethasone inducible transgenic plant line expressing AvrRpm1, and found that there appears to be no alteration in ribosylation state due to the presence of AvrRpm1 (Figure S5). To confirm that we were indeed assaying for ribosylation we treated duplicate cellular extracts with phosphodiesterase, which cleaves the ester bond in the ADP-ribose moiety, freeing the biotin label (Figure S5A). This ribosylated band appears to be of the same molecular weight as the endogenously ribosylated protein identified by Wang *et al.* in April 2011 [65]. We also tested for direct activity on RIN4, as well as any alteration in ribosylation signatures between wild type AvrRpm1 and D185A using a transient expression system in *N. benthamiana* [12], but again observed only what appeared to be an endogenous ribosylation event (Figure S5C). In the absence of additional functional tests, it also remains plausible that AvrRpm1 functions as a molecular mimic of an ADP-ribosyl transferase, and blocks that enzyme's function.

## Discussion

We demonstrate that the type III effector protein AvrRpm1 displays a homologous fold to ADP-ribosyl transferases (Figure 1A and B). This homology extends to residues (H63-Y122-D185) that are required for both AvrRpm1 virulence and recognition of AvrRpm1 by the NB-LRR protein RPM1 (Figure 2A). Mutation of the putative catalytic residue D185 results in a unique loss of virulence phenotype on susceptible plants; this phenotype is suppressed when this protein is mislocalized (Figures 3A and S3). This phenotype reflects the recognition of AvrRpm1_D185A_ by the NB-LRR protein RPS2 (Figure 3B). Additionally, this phenotype is not altered by the absence of RIN4 (Figure 3C) and the missense alleles do not contribute to an increase in interference with AvrRpt2 cleavage of RIN4 (Figure 4).

The poly(ADP-ribosyl)ation reaction in mammalian systems is involved in stress signaling, chromatin modulation, transcriptional regulation, proteasome activation and cell death [28]. ADP-ribosylation is employed by microbes to manipulate eukaryotic host cell signaling machinery. Diphtheria toxin from *Corynebacterium diphtheriae* and ExoS from *Pseudomonas aeruginosa* are virulence effectors for pathogens of mammals that target elongation factor 2 (eEF-2) and Ras GTPase, respectively [53].

There is growing evidence that ADP-ribosylation plays a critical role in phytopathogenicity as well as in plant immune responses. While no biochemical function has been attributed to *P. syringae* AvrPphF/HopF1, this effector adopts a fold similar to members of the Diphtheria toxin family of ADP-ribosyl transferases. Missense mutation of the catalytic histidine and glutamate residues led to both loss of the ability to trigger efficient disease resistance on resistant bean cultivars, and a decrease in pathogen growth on susceptible bean cultivars [66], analogous to our findings. Despite the inability to assign a biochemical function to HopF1, the homologous type III effector HopF2 has been shown to possess ADP-ribosylation activity on MAP kinase kinase 5, leading to inhibition of MTI [22]. Additionally HopU1, which is structurally similar the Cholera toxin family, is able to catalyze the addition of ADP-ribose onto the glycine rich RNA-binding protein GRP7, a component of plant innate immunity [20], [33].

Beyond direct targeting of ADP-ribosyl transferase toxins to host substrates, it appears that the host ADP-ribosyl transferase pathway itself is activated during immune response: genes encoding both PARG and Nudix hydrolases are up-regulated in the presence of MAMPs, in particular the flagellar peptide-flg22 [30]. Given these host responses to MAMPs, it is plausible that non-functional ADP-ribosyl transferase proteins could also interfere with wild type cellular outputs, as we postulate may occur for AvrRpm1. Hence, our results coincide with the increasing evidence that the ADP-ribosylation pathway plays a critical role in the interplay between phytopathogen and host.

AvrRpm1 appears to function as a molecular mimic of ADP-ribosyl transferases; whether it has enzymatic activity remains unknown. We could not determine a biochemical function for AvrRpm1, despite our demonstrated genetic requirement for intact residues analogous to those forming the PARP catalytic triad. We attempted to purify both wild type AvrRpm1 and AvrRpm1_D185A_, as well as the most divergent allele *P. syringae* pv. *phaseolicola* 2708 from various sources. In all cases, the homogenous protein we recovered did not maintain folded conformation. To side-step these challenges, we utilized transgenic plant lines conditionally expressing AvrRpm1 and modified the ADP-ribosylation assays presented in Wang *et al.* [22] to define its biochemical function and potential substrate(s). However, we did not observe increased ADP-ribosylation compared to background levels (Figure S5). Additionally the band that is ADP-ribosylated in this assay is approximately the same size as a band that was identified previously as an endogenously ADP-ribosylated protein in Arabidopsis [65]. We cannot rule out that our current assays are below detection threshold for identification of AvrRpm1 ADP-ribosylation activity. Additionally, the transient nature of the reaction and its reversal by PARG may interfere with accurately capturing targets in a whole cell context. Further work will need to be done on isolation of AvrRpm1 and identification of target proteins so that direct biochemical analysis may be performed.

Chisholm *et al.* [16] proposed that AvrRpm1 evolved to block RIN4-mediated MAMP defenses, and that this led to the evolution of RPM1. In their model, AvrRpt2 evolved to overcome AvrRpm1-dependent activation of RPM1, and RPS2 evolved to prohibit its function. Both RPM1 and RPS2 demonstrably monitor the integrity of RIN4 [6], [7], [15]. However, the work presented above and previous findings show (1) that RIN4 is dispensable for AvrRpm1 and AvrRpt2 virulence function [15], [37]; (2) that both RPM1 and RPS2 can be activated by over-expression of wild type AvrRpm1 [7], [36]; and (3) that AvrRpm1 and AvrRpt2 are, to date, never found in the same strain [6], [7], [13], [15], [36], [64], [67].

Importantly, Kim *et al.* [36] demonstrated that over-expression of AvrRpm1 can activate RPS2 in *rpm1* plants; this activation was not directly attributed to an alteration in the phosphorylation state of RIN4. They hypothesized that what were once believed to be phenotypic cytotoxic indicators of ‘effector virulence activities’ are actually the phenotypes of weak ETI [36]. Our work supports this finding, in that RPS2 is activated by the putatively catalytic missense allele AvrRpm1_D185A_ delivered at near wild type levels from *P. syringae* or *P. flourescens*.

This surprising result could be due to several factors. First, AvrRpm1 is likely to have multiple homologous targets within the host [15], as with AvrPto and AvrPphB targeting multiple receptor kinases and receptor-like kinases [68], [69], or AvrRpm1 may target multiple unrelated proteins analogous to HopF2's activity on both RIN4 and MEKK5 [9], [22]. Second, AvrRpm1_D185A_ could either bind more tightly to, or be less able to release from, an RPS2-associated target protein than the wild type AvrRpm1. Our results reinforce the evidence that RIN4 is not the only AvrRpm1 target in Arabidopsis [15], [70], and re-focus attention onto the other ten members of the RIN4-like NOI-domain containing proteins in Arabidopsis [18], [64]. RPS2 exists in a lipid raft with other components that contribute to immune signaling, potentially including RPM1 and the flagellin receptor FLS2, and can be cross linked to these components [71]. Thus, there exists the intriguing possibility that RPS2 can also monitor perturbation of alternative target(s). Experimental analysis of this idea is difficult, since *rin4* mutation is lethal in the presence of RPS2 [6]. Immune signaling in Arabidopsis may thus function like “bells on a string”- when one part of the signaling complex gets perturbed the rest makes a sound with the amplitude and relative “pitch” modified by the composition of the signaling complex, even in the absence of what was previously believed to be the major component, as we observed for weak activation of RPS2 in the absence of RPM1.

## Supporting Information

Figure S1 **Alignment of AvrRpm1 alleles.** Alignment of AvrRpm1 alleles generated with ClustalX. Conserved regions between PARP and AvrRpm1 are highlighted in light blue.
(TIF)

Figure S2 ***Pto*** **DC3000 expressing AvrRpm1 missense mutations cannot grow on wild type plants.** Two week old Col-0 seedlings were dipped into an inoculum with 10^5^ cfu/mL *Pto* DC3000 carrying either an empty vector or *avrRpm1* with missense mutations eliminating localization to the membrane (G2A) [11], or in putative catalytic triad (Y122A and D185A) and a double mutant (G2A D185A). Samples were assayed for bacterial growth on day 0 and day 3. Error bars represent 2× SEM.
(TIF)

Figure S3 **A mislocalized AvrRpm1 double mutant, G2A D185A does not limit virulence.** Growth of *Psm* CR299 (carrying an insertion in *avrRpm1*) was complemented in *trans* with *avrRpm1* and the indicated missense mutations. Leaves of 4-week-old *rpm1* plants were inoculated with 10^6^ cfu/mL and samples were collected on day 0 and day 3. Error bars represent 2× SEM. An analysis of variance (ANOVA) was performed among the day 3 samples followed by Tukey's post-hoc analysis (α = 0.05) with significance groups indicated by letters on the graph.
(TIF)

Figure S4 **Generation of new antibody using RIN4 specific peptide.** New antibody against RIN4 was generated against peptide from amino acids 57 to 69 (PSSRTKPEQVDTV) based on high antigenicity and sequence uniqueness. Immunoblot analysis was performed on wild type (Col-0) and plants lacking RIN4 protein (*rpm1 rps2 rin4*).
(TIF)

Figure S5 **AvrRpm1 does not preferentially ribosylate Arabidopsis proteins, or RIN4.** (**A**) Two week old seedlings were sprayed with a solution of 25 mM dexamethasone and 25 nM biotinylated NAD. Seedlings were collected 12 hours later and a duplicate sample was treated with PDE type I to remove the ribosylation modification. Samples were then subjected to immunoblot analysis with α-streptavidin antibody. (**B**) Replicate samples as in (**A**) subjected to immunoblot analysis α-HA antibody for expression of AvrRpm1-HA. (**C**) *N. benthamiana* was left un-infiltrated or was infiltrated with *A. tumefaciens* carrying RIN4 and either estradiol inducible AvrRpm1-HA or AvrRpm1_D185A_-HA. Upon induction of AvrRpm1 expression, leaves were also treated with biotinylated NAD and six hours later samples were collected and subjected to immunoblot analysis. Figure shows expected apparent molecular weight range for RIN4 (23 kDa). (**D**) Replicate samples as in part (**C**) subjected to immunoblot analysis α-HA antibody for expression of AvrRpm1_WT_ and AvrRpm1_D185A_ and α-T7 antibody for expression of RIN4.
(TIF)
