# Supplementary figures and images for: AvrRpm1 Missense Mutations Weakly Activate RPS2-Mediated Immune Response in Arabidopsis thaliana
Source: PLoS One. 2012 Aug 6;7(8):e42633. doi: 10.1371/journal.pone.0042633 (PMC3412798; doi:10.1371/journal.pone.0042633)

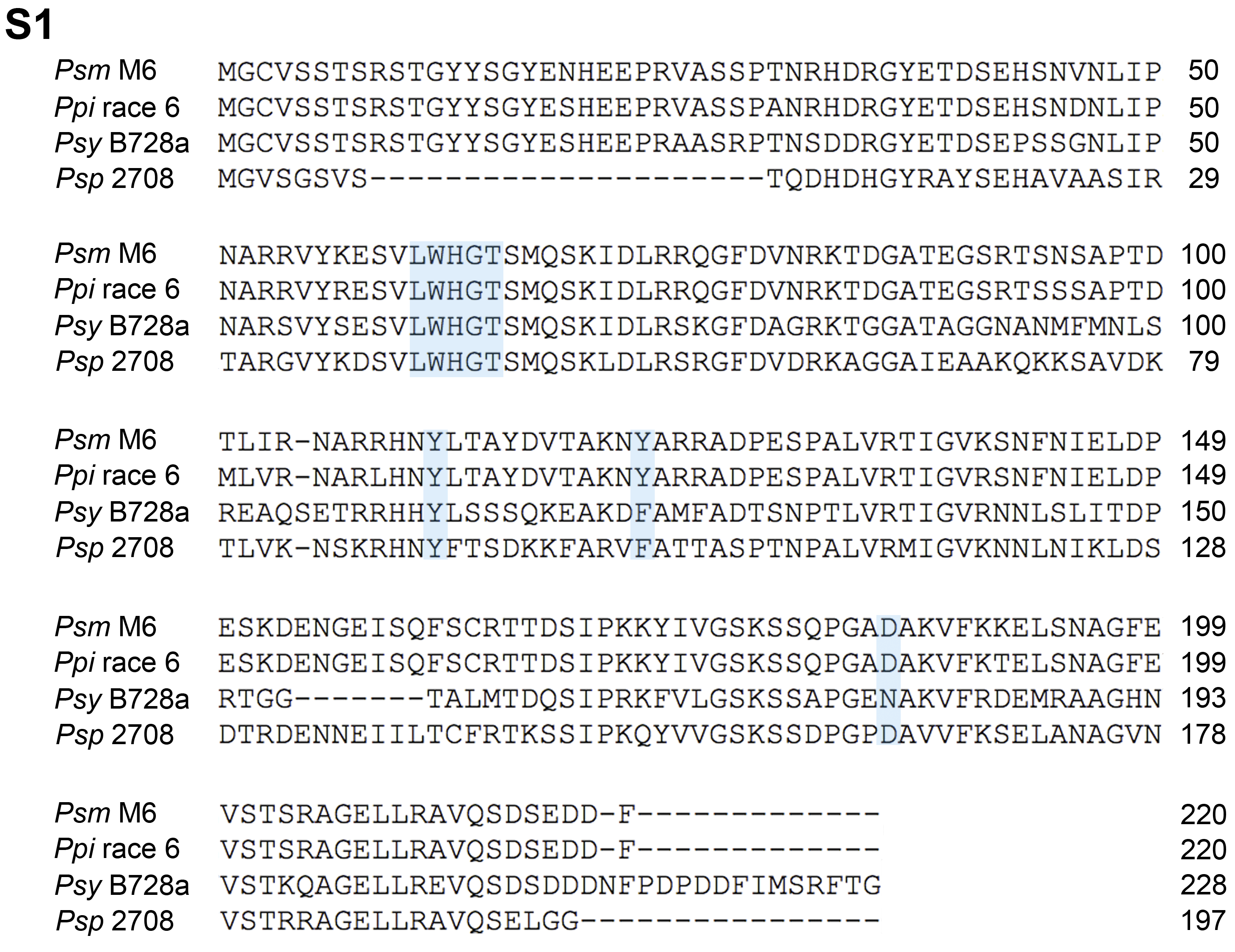

Supplement: Figure S1 — Alignment of AvrRpm1 alleles. Alignment of AvrRpm1 alleles generated with ClustalX. Conserved regions between PARP and AvrRpm1 are highlighted in light blue. (TIF) [file pone.0042633.s001.tif]

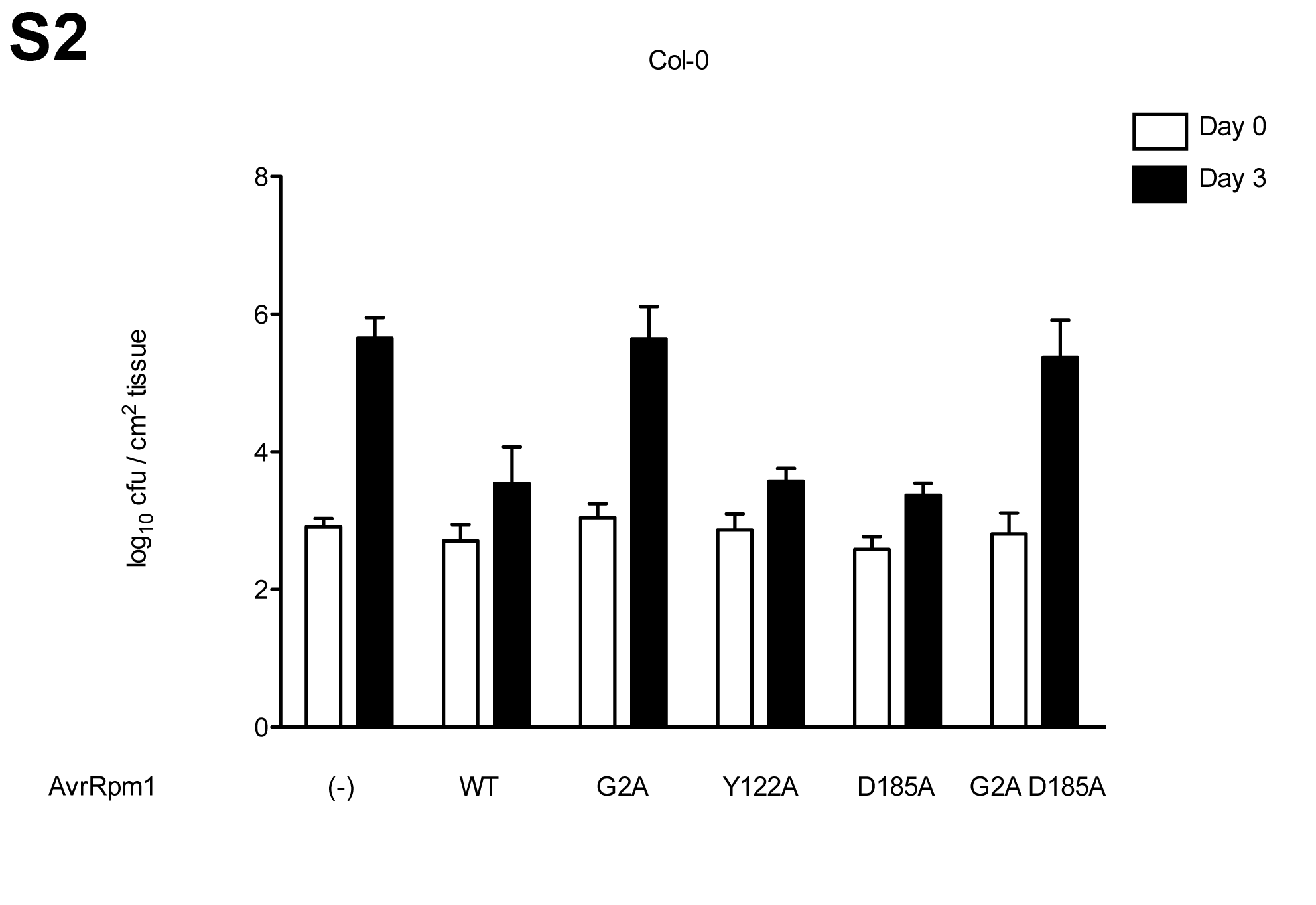

Supplement: Figure S2 — Pto DC3000 expressing AvrRpm1 missense mutations cannot grow on wild type plants. Two week old Col-0 seedlings were dipped into an inoculum with 105 cfu/mL Pto DC3000 carrying either an empty vector or avrRpm1 with missense mutations eliminating localization to the membrane (G2A) [11], or in putative catalytic triad (Y122A and D185A) and a double mutant (G2A D185A). Samples were assayed for bacterial growth on day 0 and day 3. Error bars represent 2× SEM. (TIF) [file pone.0042633.s002.tif]

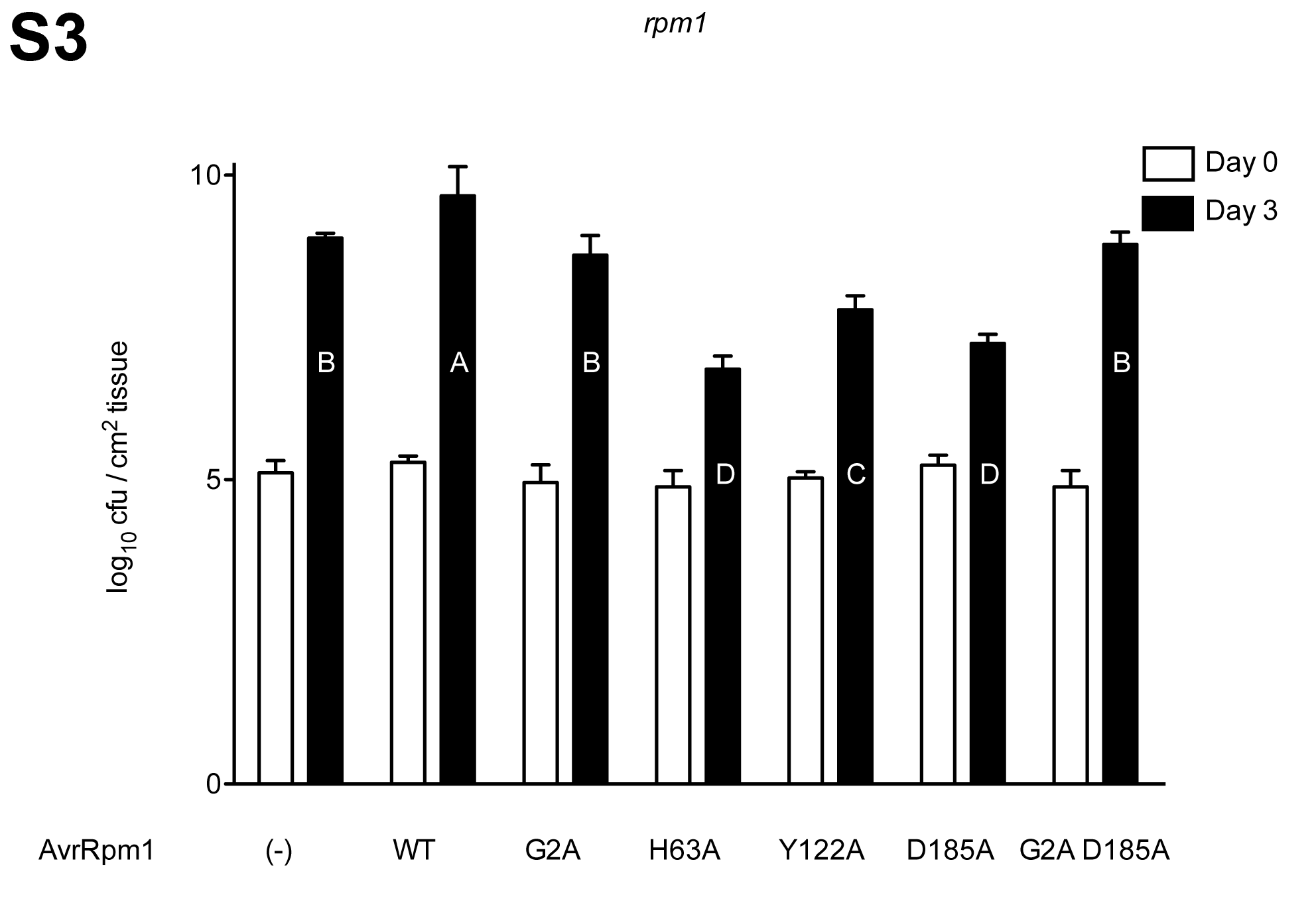

Supplement: Figure S3 — A mislocalized AvrRpm1 double mutant, G2A D185A does not limit virulence. Growth of Psm CR299 (carrying an insertion in avrRpm1) was complemented in trans with avrRpm1 and the indicated missense mutations. Leaves of 4-week-old rpm1 plants were inoculated with 106 cfu/mL and samples were collected on day 0 and day 3. Error bars represent 2× SEM. An analysis of variance (ANOVA) was performed among the day 3 samples followed by Tukey's post-hoc analysis (α = 0.05) with significance groups indicated by letters on the graph. (TIF) [file pone.0042633.s003.tif]

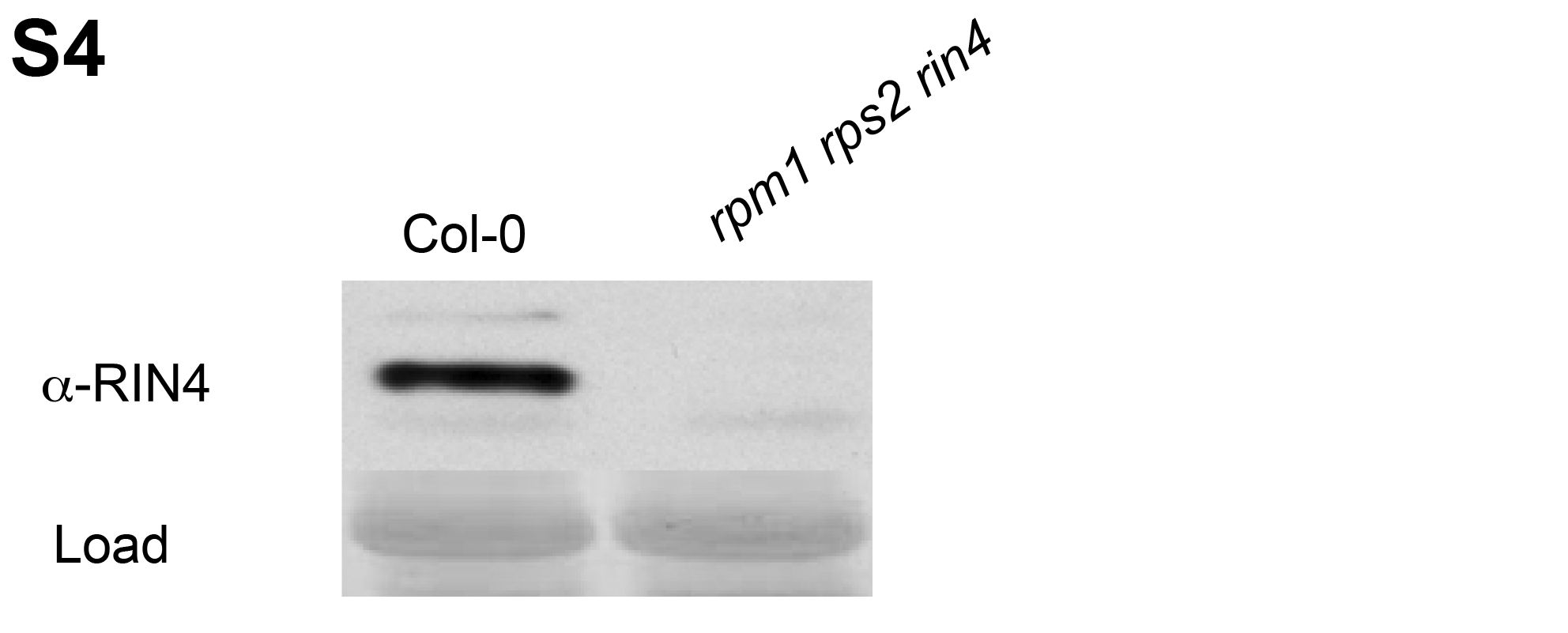

Supplement: Figure S4 — Generation of new antibody using RIN4 specific peptide. New antibody against RIN4 was generated against peptide from amino acids 57 to 69 (PSSRTKPEQVDTV) based on high antigenicity and sequence uniqueness. Immunoblot analysis was performed on wild type (Col-0) and plants lacking RIN4 protein (rpm1 rps2 rin4). (TIF) [file pone.0042633.s004.tif]

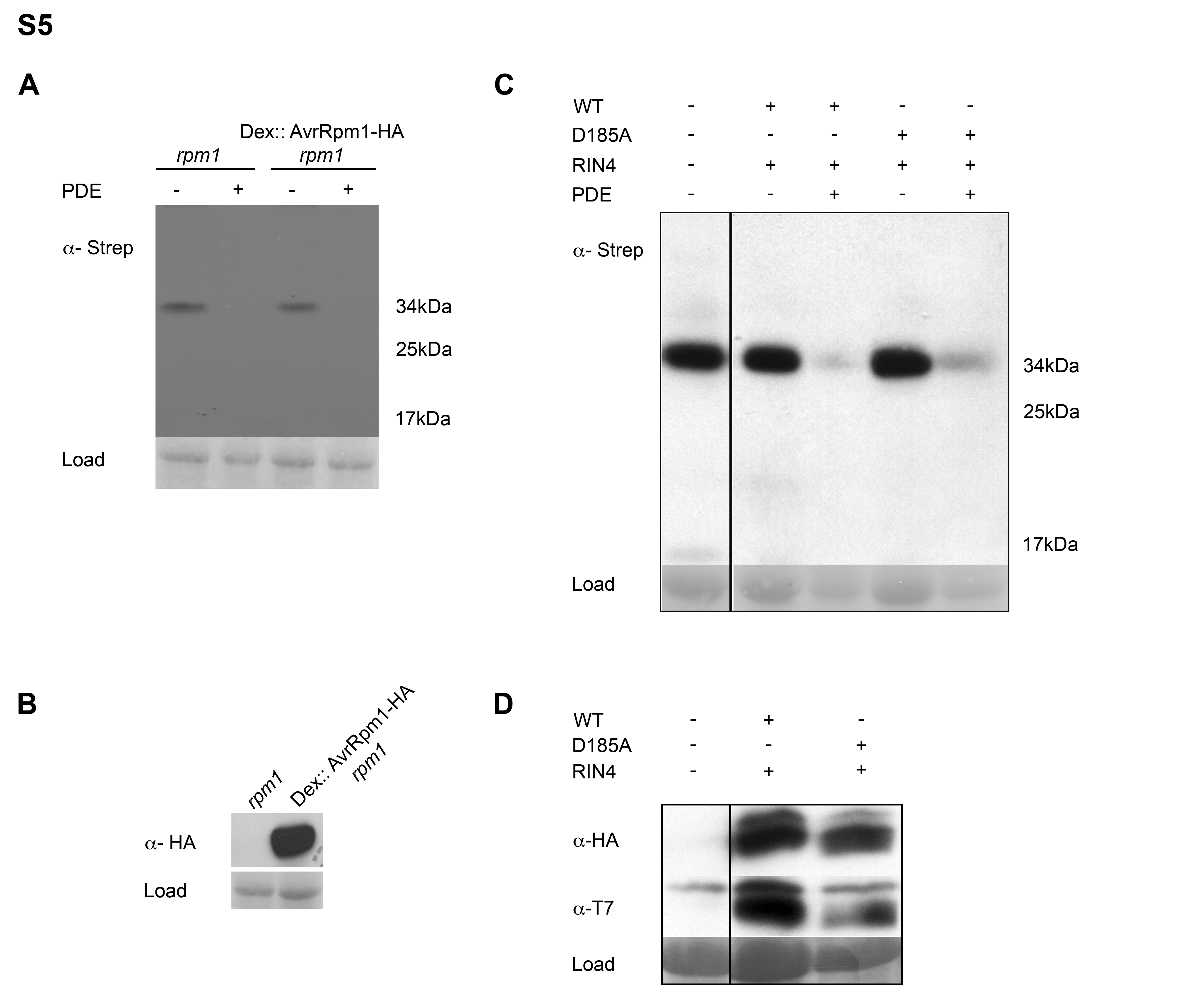

Supplement: Figure S5 — AvrRpm1 does not preferentially ribosylate Arabidopsis proteins, or RIN4. (A) Two week old seedlings were sprayed with a solution of 25 mM dexamethasone and 25 nM biotinylated NAD. Seedlings were collected 12 hours later and a duplicate sample was treated with PDE type I to remove the ribosylation modification. Samples were then subjected to immunoblot analysis with α-streptavidin antibody. (B) Replicate samples as in (A) subjected to immunoblot analysis α-HA antibody for expression of AvrRpm1-HA. (C) N. benthamiana was left un-infiltrated or was infiltrated with A. tumefaciens carrying RIN4 and either estradiol inducible AvrRpm1-HA or AvrRpm1D185A-HA. Upon induction of AvrRpm1 expression, leaves were also treated with biotinylated NAD and six hours later samples were collected and subjected to immunoblot analysis. Figure shows expected apparent molecular weight range for RIN4 (23 kDa). (D) Replicate samples as in part (C) subjected to immunoblot analysis α-HA antibody for expression of AvrRpm1WT and AvrRpm1D185A and α-T7 antibody for expression of RIN4. (TIF) [file pone.0042633.s005.tif]
